# Delayed Tension Pneumocephalus following Gunshot Wound to the Head: A Case Report and Review of the Literature

**DOI:** 10.1155/2016/7534571

**Published:** 2016-12-15

**Authors:** Arthur Wang, Elena Solli, Nathan Carberry, Virany Hillard, Adesh Tandon

**Affiliations:** Department of Neurosurgery, New York Medical College, Westchester, Valhalla, NY, USA

## Abstract

Tension pneumocephalus is a rare complication of head trauma and neurosurgical procedures, amongst other causes. It is defined by the combination of intracranial air, increased intracranial pressure, and mass effect. Although it often presents soon after surgery, it can also rarely present in a delayed fashion. We present a case of delayed tension pneumocephalus, occurring approximately 16 weeks after bifrontal craniectomy for a self-inflicted gunshot wound. Following a month of rhinorrhea, postnasal drip, and cough, the patient presented with a sensation of expansion in the area of the right forehead. As tension pneumocephalus is an emergency that can be fatal, this patient was treated expediently and avoided severe neurological deficits. The case recounted here is important as a demonstrative example that tension pneumocephalus does not always follow a defined course immediately after trauma or neurosurgery but rather can develop insidiously without obvious signs.

## 1. Introduction

Pneumocephalus is defined as a collection of air in the intracranial cavity [1]. It is commonly observed in association with neurosurgical intervention, tumors, radiation, infection, and trauma [2]. Trauma is the most common cause of pneumocephalus, representing the etiology in 75% of cases [3]. However, pneumocephalus is found in approximately 0.5–1.0% of cases of head trauma overall [4]. In particular, it is associated with fractures of the basal skull, paranasal sinuses, and open cranial convexities [1]. Pneumocephalus is also commonly found after a variety of neurosurgical procedures, particularly those involving the intracranial space, sinuses, orbits, or nasal passages [4]. It is seen in up to 100% of cases involving supratentorial craniotomy, as a certain amount of air in the intracranial cavity is normal after this procedure [1, 5, 6]. It can also occur after creation of burr holes, placement of VP shunts, lumbar puncture, nasal septum resection, nasal polypectomy, or sinus surgery using transsphenoidal or endoscopic approaches [1, 5, 7]. In addition, other potential iatrogenic causes have been described, including nitrous oxide anesthesia, continuous positive pressure ventilation, hyperbaric oxygen therapy, barotrauma, spinal anesthesia, ICP monitoring, and intraoperative mannitol [1, 3]. Radiation therapy may also lead to pneumocephalus postoperatively, as it interferes with dural healing [8]. It is also known to occur in association with other intracranial pathologies, such as infection with gas-producing bacteria, and tumor.

Tension pneumocephalus, an entity distinct from typical pneumocephalus, is defined as pneumocephalus that results in intracranial hypertension and mass effect [3]. It may eventually lead to neurological symptoms, including signs of herniation, and is life threatening [2]. Although some amount of pneumocephalus after neurosurgical procedures is common, tension pneumocephalus is much more rare and is observed in only 0.1–0.2% of craniotomies [5, 7]. It is important to distinguish benign postsurgical pneumocephalus from tension pneumocephalus because the latter is a neurosurgical emergency [9].

## 2. Case Report

### 2.1. History and Examination

**Figure 1:**
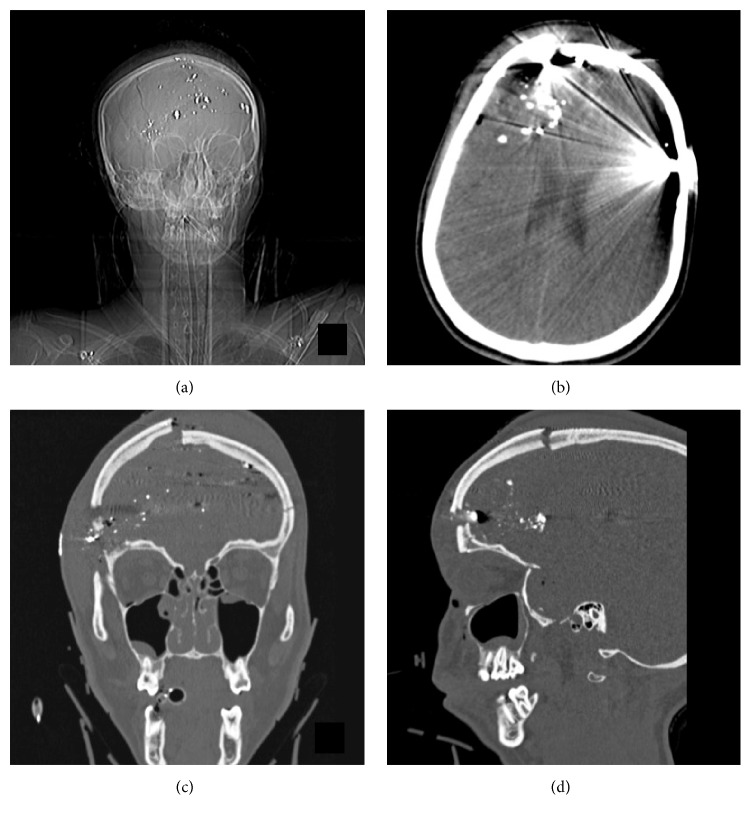
Preoperative computed tomography (CT) of the head and neck. (a) Scout image demonstrating multiple metallic fragments intracranially and in the bifrontal soft tissue and bone. (b) Axial noncontrast CT demonstrating an extensive right frontal skull fracture with intracranial acute blood products including intraparenchymal, subarachnoid, subdural, and epidural components. (c, d) Coronal and sagittal reconstructions demonstrating the entry and exit sites of the bullet and the comminuted displaced free floating right frontal bone fragment.

Our patient, in the second decade of life, with an extensive history of depression and polysubstance abuse presented to our institution after sustaining a self-inflicted gunshot wound with a .22 caliber bullet. At the scene, the patient had a Glasgow Coma Scale Score (GCS) of 14. In the trauma bay, her neurological exam rapidly declined and she was intubated for airway protection. A computed tomography (CT) of the head demonstrated a right frontal bullet entry site with a severe comminuted displaced right frontal bone fracture (Figures 1(a), 1(c), and 1(d)). Shattered bullet fragments are seen in the soft tissue and bone. Intracranially, there was extensive gas and acute blood products including intraparenchymal, subarachnoid, subdural, and epidural hemorrhage (Figure 1(b)). A maxillofacial-sinus CT demonstrated a small right frontal sinus fracture, maxillary sinus, and orbital fractures. The patient was taken to the operating room and underwent a bifrontal craniectomy and evacuation of the bifrontal intracerebral hematoma. Intraoperatively, no evidence of frontal sinus injury was detected. However, the dura was lacerated through the trajectory of the bullet which was addressed by a primary dural repair and duraplasty with Duragen dural allograft (Integra Lifesciences, Plainsboro, New Jersey, USA). An external ventricular drain was placed for CSF drainage and intracranial pressure monitoring in the intensive care unit.

Postoperatively, the patient did not complain of any rhinorrhea or otorrhea and a discharge head CT did not demonstrate any pneumocephalus. She was discharged home with a helmet.

**Figure 2:**
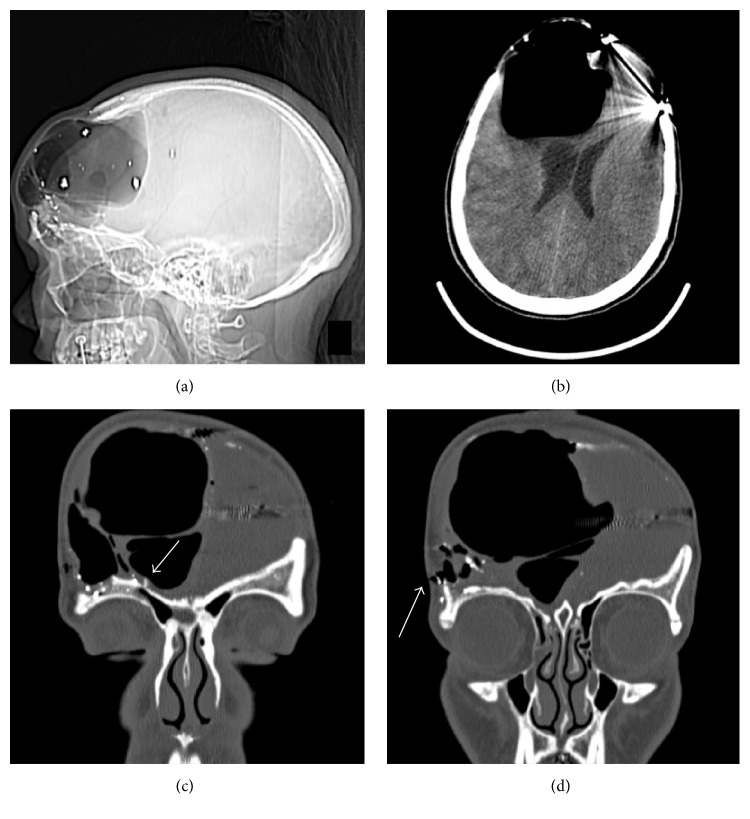
Readmission CT head and maxillofacial-sinus scan. (a) Scout image demonstrating a large pocket of hypodensity in the right frontal region consistent with pneumocephalus. (b) Axial CT demonstrating right frontal tension pneumocephalus with significant mass effect including effacement of the right lateral ventricle, midline shift, and subfalcine herniation. (c, d) Coronal reconstructed images demonstrating air communicating through the skin in the right orbital-frontal region and a small defect with shard of bone in the superior aspect of the right frontal sinus.

16 weeks after discharge, the patient presented in the emergency department with complaints of chronic headaches, postnasal drip associated with a salty taste, and a recent episode of “sudden expansion” of her right forehead at the site of the craniectomy. On examination, CSF rhinorrhea was determined when active drainage of clear fluid was seen from her right nostril when her head was placed in flexion. Her scalp flap was very full but soft. The patient had a low grade fever but all labs were within normal limits. A CT head demonstrated tension pneumocephalus in the right frontal lobe with significant mass effect including effacement of the right lateral ventricle, midline shift, and subfalcine herniation (Figures 2(a) and 2(b)). Coronal maxillofacial-sinus reconstructed images demonstrated air communicating through a laceration of the skin in the right orbital-frontal region and a small defect with a shard of bone in the superior aspect of the right frontal sinus (Figures 2(c) and 2(d)). A contrast based CT head did not demonstrate an intracranial abscess.

Given the constitution of findings, the patient was placed supine and started on 100% oxygen and broad spectrum antibiotics.

### 2.2. Operation and Findings

We planned a bicoronal approach for repair of the frontal sinus repair, repair of CSF leak, and cranioplasty with retrieval of bone flap. After reflecting the scalp flap anteriorly to the superior orbital rims, we dissected down to the region of the anterior cranial fossa where we visualized three small defects in the anterior cranial fossa leading to the frontal sinus. We packed the frontal sinus with small amounts of bone chips and harvested galeal free graft and applied a layer of Co-Seal surgical sealant (Baxter Healthcare, Freemont, California, USA). Upon opening the dural allograft, we evacuated a copious amount of pneumocephalus and repaired a small dural defect from where CSF extravasation was seen. Dural allograft and Co-Seal were utilized to seal this area. After verifying no CSF egress with multiple Valsalva maneuvers, the bone flap was fixated to her skull.

### 2.3. Postoperative Course

**Figure 3:**
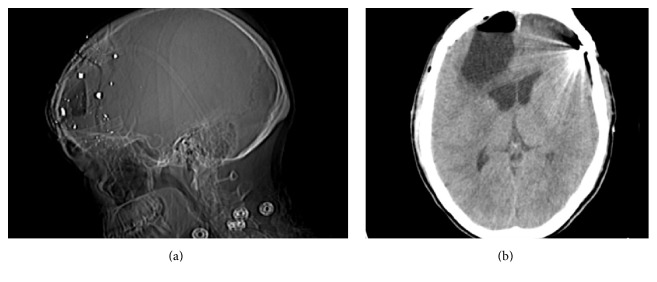
Postcranioplasty CT head. (a) Scout image showing reduction in size of pneumocephalus. (b) Axial noncontrast head CT demonstrates marked resolution of pneumocephalus and decreased midline shift and mass effect on the right lateral ventricle.

The patient remained in the intensive care unit immediately postoperatively. The patient was kept in bed rest for 3 days before we slowly mobilized her. No spinal drainage was used or required in this case. She recovered well from the procedure. An exit CT head was performed prior to discharge showing decreased pneumocephalus (Figure 3). The patient was discharged 6 days after surgery. Her antibiotics were stopped before discharge.

## 3. Discussion

The underlying pathophysiology of tension pneumocephalus depends on the presence of an aberrant pathway through which air can enter the cranium and a factor that promotes air inflow [5]. Two possible mechanisms for the development of tension pneumocephalus have been described. The “ball-valve” theory, described by Dandy in 1926, postulates that extracranial air enters the cranium via a dural defect that acts as a valve, allowing air to enter in one direction but preventing its exit [1, 3, 7]. When extracranial and/or nasopharyngeal pressure is altered, as occurs during coughing or sneezing, air may be pushed intracranially through this kind of dural defect [4, 6]. In 1964, Horowitz described the “inverted soda bottle effect,” which postulates that negative intracranial pressure created by a CSF leak may draw air into the intracranial cavity, as the air replaces the lost fluid volume via a pressure gradient between the extracranial and intracranial compartments [1, 6, 7, 10, 11]. Another contributing factor may include the thermal expansion of air by body heat, which increases the volume of air that remains in the cranial cavity [7]. Air may also enter the cranium directly via penetrating trauma or by the production of gas inside the head by infection with certain bacteria [4].

This case report offers a few interesting points worth discussing. Our patient had a very delayed onset of tension pneumocephalus, nearly 15 weeks after her surgery. At the time of her bifrontal craniectomy, the frontal sinus was never entered and no injury was seen. In addition, no CSF rhinorrhea or otorrhea was ever noted during her hospital stay. On discharge head CT, no pneumocephalus was seen. Despite all this, the patient represented with rhinorrhea and tension pneumocephalus. Her delayed tension pneumocephalus can be explained in two ways. First, the patient had a small defect in the right frontal sinus on admission maxillofacial-sinus CT which when combined with her dural laceration would have led to her rhinorrhea on second admission. This CSF leak would allow air to collect in a similar mechanism as how air enters an inverted bottle of water as the fluid escapes. The dural breach allows CSF to escape which creates a negative intracranial pressure for air to enter. Second, her delayed neurological decline is likely related to her craniectomy defect which affords room for significant pneumocephalus to collect. Any patient with an intact cranium would be unable to handle such an amount of gas and would present at an earlier time.

This case also highlights the importance of excluding an intracranial infection. Pneumocephalus can occur secondary to gas-producing organisms in the cranium. It is important to rule out an intracranial abscess in these patients. Given our patient's history of intracranial foreign objects, new onset rhinorrhea, and low grade temperatures, broad spectrum antibiotics were initiated while waiting for a contrast head CT to rule out intracranial abscess. In the setting of CSF leak and clinical signs of infection, we recommend starting broad spectrum antibiotics as the radiographic workup is conducted.

The literature on tension pneumocephalus is limited to a handful of case reports and small case series. Although it commonly presents acutely, it is possible for pneumocephalus to present in a delayed fashion. Delayed pneumocephalus is defined by some authors as occurring at greater than 72 hours after the initial head trauma [3]. However, a few case reports in the literature have shown that pneumocephalus can present even later. Sprague and Poulgrain described a case of delayed tension pneumocephalus presenting 11 days after craniotomy for resection of an anterior skull base meningioma in which the frontal sinus was entered [11]. Demetriades et al. described another case of delayed tension pneumocephalus, presenting 5 weeks after a craniotomy for resection of a right frontotemporal tumor [8]. In yet another case, tension pneumocephalus presented seven years after a craniotomy for clipping of an anterior communicating artery aneurysm [5].

Because tension pneumocephalus mimics the behavior of a space occupying mass, the clinical presentation of these patients includes headache, confusion, lethargy, and focal weakness [5, 8, 11].

The head CT findings in our patient are not typical of those seen in classic tension pneumocephalus. On CT scan, one may observe the characteristic* Mount Fuji sign*, which was described initially by Ishiwata [1]. This sign is pathognomonic for tension pneumocephalus. It appears on CT as bilateral subdural hypoattenuations with a widened interhemispheric fissure, representing the compression and separation of the frontal lobes by subdural air [1, 9]. One may also observe bilateral frontal lobe compression, without the characteristic separation of the frontal lobes found in* Mount Fuji sign*; this has been termed the* peaking sign* [3]. However, tension pneumocephalus can occur in any compartment in the cranium, including the epidural, subdural, subarachnoid, and intraventricular compartments [4]. Epidural air is seen on CT as a biconvex area of hypoattenuation that does not change with movement. Subdural air outlines the inside of the skull and changes with movement. Subarachnoid air is visualized as areas of hypoattenuation under the sulci and cisterns or can be seen in the ventricles. Intracerebral air can be seen as areas of hypoattenuation surrounded by brain parenchyma [4]. Ishiwata described the* air bubble sign*, which is air within the cisterns, which can also lead to pneumocephalus [7]. Our patient presented with a focal intracerebral collection of gas that resembled a space occupying lesion. This radiographic presentation should be recognized in future cases of tension pneumocephalus.

Treatment of tension pneumocephalus varies based on the severity of the condition. In general, most cases of pneumocephalus resolve with conservative treatment. Isolated pneumocephalus without neurologic signs can be treated with measures that include maintained head elevation or placement of patient in Fowler's position (head of bed elevation at 30°); administration of osmotic diuretics, analgesics, and antipyretics; and avoidance of any maneuvers that may increase intracranial or sinus pressure such as Valsalva [3, 4]. Administration of 100% oxygen has also been found to increase the rate of reabsorption of pneumocephalus [2]. The relatively small amounts of air that are commonly found after craniotomy typically reabsorb spontaneously within 3-4 weeks and do not cause symptoms or clinical sequelae [12].

However, in cases of tension pneumocephalus that involve significant intracranial hypertension, emergent decompression is indicated as the condition can be fatal. Treatment options include the placement of a burr hole, needle aspiration, craniotomy, and placement of a subdural drain or external ventricular drain [2–4, 9]. Once the air is evacuated, any dural defect that may have allowed air to enter the cranium must be repaired, as this is the only way to definitively resolve the condition [11]. It is rare for a case of asymptomatic large volume pneumocephalus to not require decompression [3].

In our patient, tension pneumocephalus occurred in the setting of an active CSF leak. Surgery was performed to both decompress the intracranial air and seal the dural and skull base defect. It is not enough to only decompress the pneumocephalus without addressing the underlying dural and skull base defect. The pneumocephalus will recur. This can be performed with a combined neurosurgery and ENT team.

Our case demonstrates how tension pneumocephalus can present in a delayed fashion following initial insult and surgery. In addition, it may present insidiously with nonspecific symptoms. Furthermore, symptoms of CSF leak may or may not be apparent at the time of initial injury or directly after surgery. This indicates how important it is to maintain an appropriate level of clinical suspicion for this condition in patients with head trauma or previous neurosurgical intervention, as this condition must be rapidly addressed.
